# Mouse Transgenesis in a Single Locus with Independent Regulation for Multiple Fluorophores

**DOI:** 10.1371/journal.pone.0040511

**Published:** 2012-07-12

**Authors:** Joseph D. Dougherty, Juliet Zhang, Huifen Feng, Shiaoching Gong, Nathaniel Heintz

**Affiliations:** 1 Laboratory of Molecular Biology, The Rockefeller University, New York, New York, United States of America; 2 Departments of Genetics and Psychiatry, Washington University School of Medicine, St. Louis, Missouri, United States of America; 3 The GENSAT project, The Rockefeller University, New York, New York, United States of America; 4 Howard Hughes Medical Institute, The Rockefeller University, New York, New York, United States of America; University College London, United Kingdom

## Abstract

A major barrier to complex experimental design in mouse genetics is the allele problem: combining three or more alleles is time-consuming and inefficient. Here, we solve this problem for transgenic animals with a simple modification of existing BAC transgenesis protocols, and generate triple-colored ‘prism’ mice in which the major cell types of the brain: neurons, astrocytes, and oligodendrocytes, are each labeled with a distinct fluorophore. All three fluorophores are expressed from the same locus, yet each fluorophore is expressed in an independent temporal and spatial pattern. All three transgenes are generally co-inherited across multiple generations with stable genomic copy number and expression patterns. This generic solution should permit more sophisticated experimental manipulations to assess functional interactions amongst populations of cell types *in vivo* in a more rapid and efficient manner.

## Introduction

Over the last three decades, transgenic mice have become a mainstay of biological research, particularly for studies of gene expression and genetic gain of function experiments [1], [2], [3]. The advent of BAC (Bacterial Artificial Chromosome) transgenesis [4], has expanded this toolkit to permit reproducible targeting of particular cell types with transgenes for experimental monitoring [5], [6], [7] and manipulation [8], [9], [10], as well as CRE-mediated cell specific genetic modification [11]. However, as experimental designs become more sophisticated, involving multiple alleles and distinct mouse backgrounds, breeding paradigms become severely rate limiting. Currently, most studies tend to utilize at most two alleles [12], [13], [14]. A consideration of simple mendelian rules highlights the reason: the allele problem. To combine three or more alleles is time-consuming, inefficient, and wasteful of animal lives and experimental resources; only one animal in eight (2^3^) from heterozygote matings would carry all three transgenes and thus be of use for experimental applications.

Likewise, traditional longitudinal anatomical studies, such as those for development or neural repair applications, require the sacrifice of a number of research animals for each time point and condition of interest. Technical advances in microscopy now permit relatively non-invasive *in vivo* longitudinal monitoring of cellular anatomy and activity, provided those cells are somehow labeled with appropriate fluorophores. However, most existing transgenic mouse lines only label one particular cell type, precluding studies entailing cellular interactions, or more comprehensive monitoring of ongoing processes.

Ideally, one would be able to generate mouse lines where multiple transgenes have been inserted into a single locus, yet where each transgene maintains independent regulation in distinct cell types. Fortunately, transgenes tend to integrate into single loci as tandem concatamers [2], [15], and it is thought that large fragments of DNA such as BACs [16], [17], [18] tend to contain all the necessary *cis* regulatory information to drive faithful and consistent expression, independent of position of integration effects. Thus an apparent solution to the allele problem may be to simply co-inject multiple BACs into the same fertilized mouse egg.

This approach, if successful, could have broad applications for investigating complex neural circuitry with optogenetic and other approaches [8], [9], *in vivo* imaging of synapse formation [19], or combinatorial approaches to targeting of specific cell types [20], [21]. Yet, it remains to be seen if each transgene will maintain its distinct expression in the context of a transgenic concatamer, and at what efficiency transgenesis would occur under these conditions. The simplest test of this approach in general would be to pick a few very distinct cell types in the nervous system and determine if they could be targeted accurately and independently using spectrally distinct fluorophores. Therefore, both for proof of principle and to potentially generate tools for anatomical studies such as those above, we undertook to create mouse lines with distinctly labeled neurons, oligodendrocytes, and astrocytes.

## Results

### Generation of Prism 1.0 Mice

First, we identified three fluorophores; Yellow Fluorescent protein (YFP) [22], Cerulean [23], and mCherry [24], which had been reported to have distinct excitation and emission spectra. YFP and Cerulean are both derivatives of the *Aequorea Victoria* GFP protein, while mCherry is a derivative of the DsRed fluorophore modified to adjust the spectral properties, stability, and to make it monomeric. Next, we co-transfected these three fluorophores in 293T cells to confirm they could be distinguished using relatively standard microscopic techniques (not shown). Finally, with PCR, we added a three prime Myc tag to Cerulean and HA tag to YFP so that these two GFP derivatives could be distinguished easily by different antibodies for Western blots.

**Figure 1:**
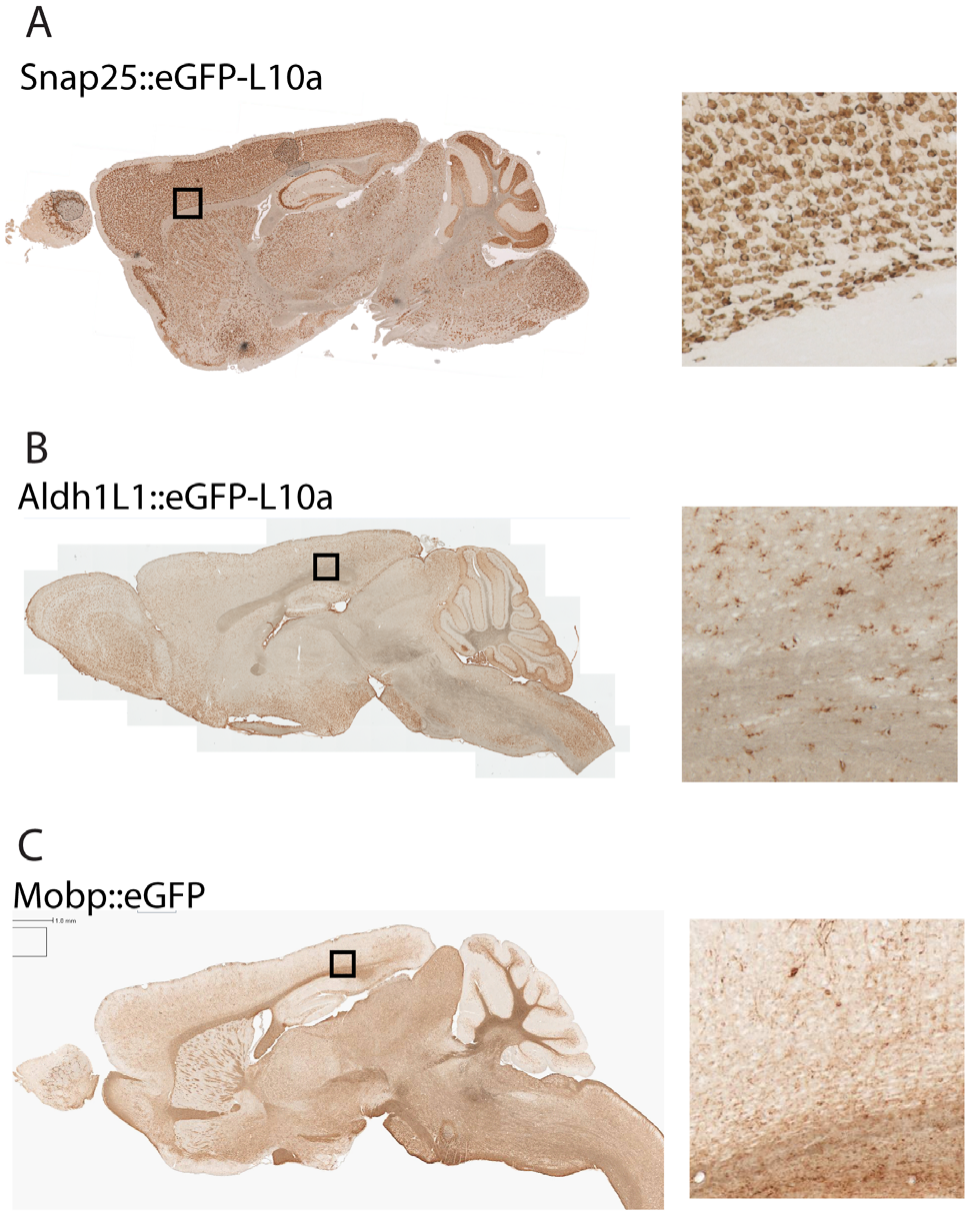
Single transgenic lines target the three major cell types of the mouse brain. A) DAB immunohistochemistry with GFP antibodies on the *Snap25* bacTRAP line exemplifies a pan-neuronal pattern of expression. B) Immunohistochemistry on *Aldh1L1* bacTRAP line exemplifies a pan-astrocytic pattern of expression. C) Immunohistochemistry on *Mobp* GENSAT [7] line exemplifies an oligodendritic pattern of expression. *Right panels: Higher magnification images of lower layers of cortex & corpus callosum. Approximate location indicated with box in left panel.*

We next identified three BACs that consistently and reliably target the three major cell types of the CNS. Synaptosomal-associated protein, 25 kDa (*Snap25*) is a component of the presynaptic machinery common to all neurons, but, based on our previous survey of gene expression in neural cells [6] is not expressed in glia. A bacTRAP line (Fig. 1A) generated using a BAC covering this locus has been confirmed to target all neurons and exclusively neurons. Aldehyde dehydrogenase 1 family, member L1 (*Aldh1L1*) is an astrocyte specific gene [25], which we have also employed previously to successfully target astrocytes (Fig. 1B) [6], [26]. Finally, we selected a BAC covering the classic marker, Myelin-associated oligodendrocyte basic protein (*Mobp*), previously shown by GENSAT to express accurately (Fig. 1C), to target oligodendrocytes [7].

To determine the feasibility of generating mouse lines with multiple independently regulated transgenes in a single locus, we engineered and prepared three separate BACs, *Mobp::YFP-HA, Aldh1L1::Cerulean-Myc,* and *Snap25::mCherry* and co-injected all three together into the pronuclei of fertilized mouse eggs. If the BACs all express independently and accurately, this should generate mice with yellow oligodendrocytes, blue-green astrocytes, and red neurons. From our first injections of 100 eggs, of the seventeen pups born, we identified by PCR three founders which had incorporated some transgenic DNA by PCR. Of these, one pup was positive for *YFP-HA(JD1864)* only, one was positive for *mCherry* and *YFP-HA(JD1861),* and the third was positive for all three transgenes*(JD1849)*. Founders were then crossed to wild-type FVB females, and F1 progeny were genotyped and processed for anatomy.

### Transgenes Express in Three Cell Types Independently and Accurately

**Figure 2:**
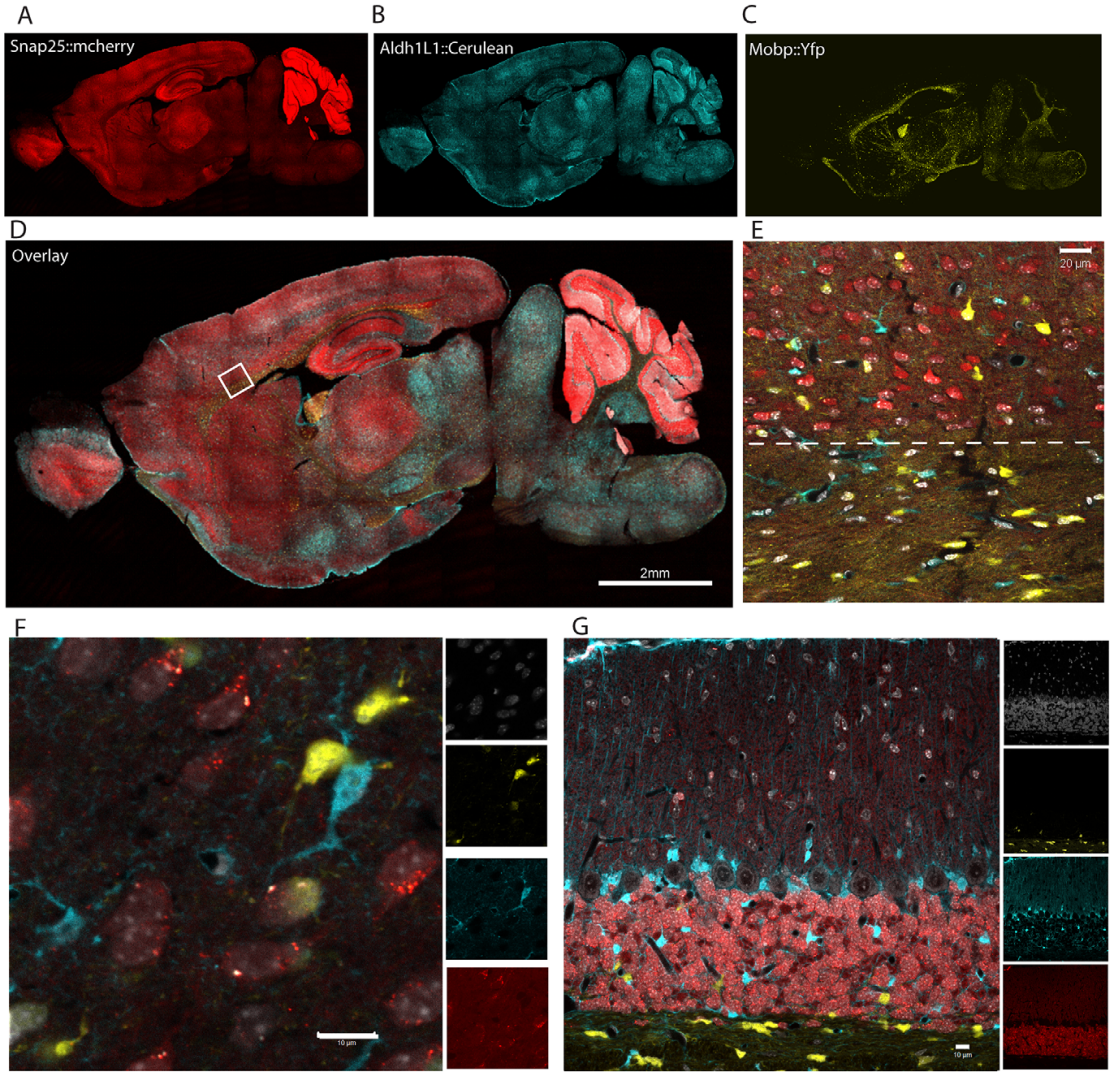
Triple transgenic mouse line targets three major cell types of the brain. A–C) Expression of three transgenes from Prism JD1849 mouse line is largely similar to patterns of single transgenic lines; A) *Snap25::mCherry,* B) *Aldh1L1::Cerulean-Myc* C) *Mobp::YFP-Ha*. D) Overlay of A–C. Box shows approximate location of E. E) Single panel showing division (dashed line) between white matter of corpus callosum (containing only oligodendrocytes and astrocytes) and gray matter of cortex (containing all three cell types). F, G) Confocal microscopy of cortex (F) and cerebellum (G) shows distinct morphology for each cell type, and lack of overlap for fluorophores. Nuclei counterstained with To-Pro-3-iodide (white).

**Figure 3:**
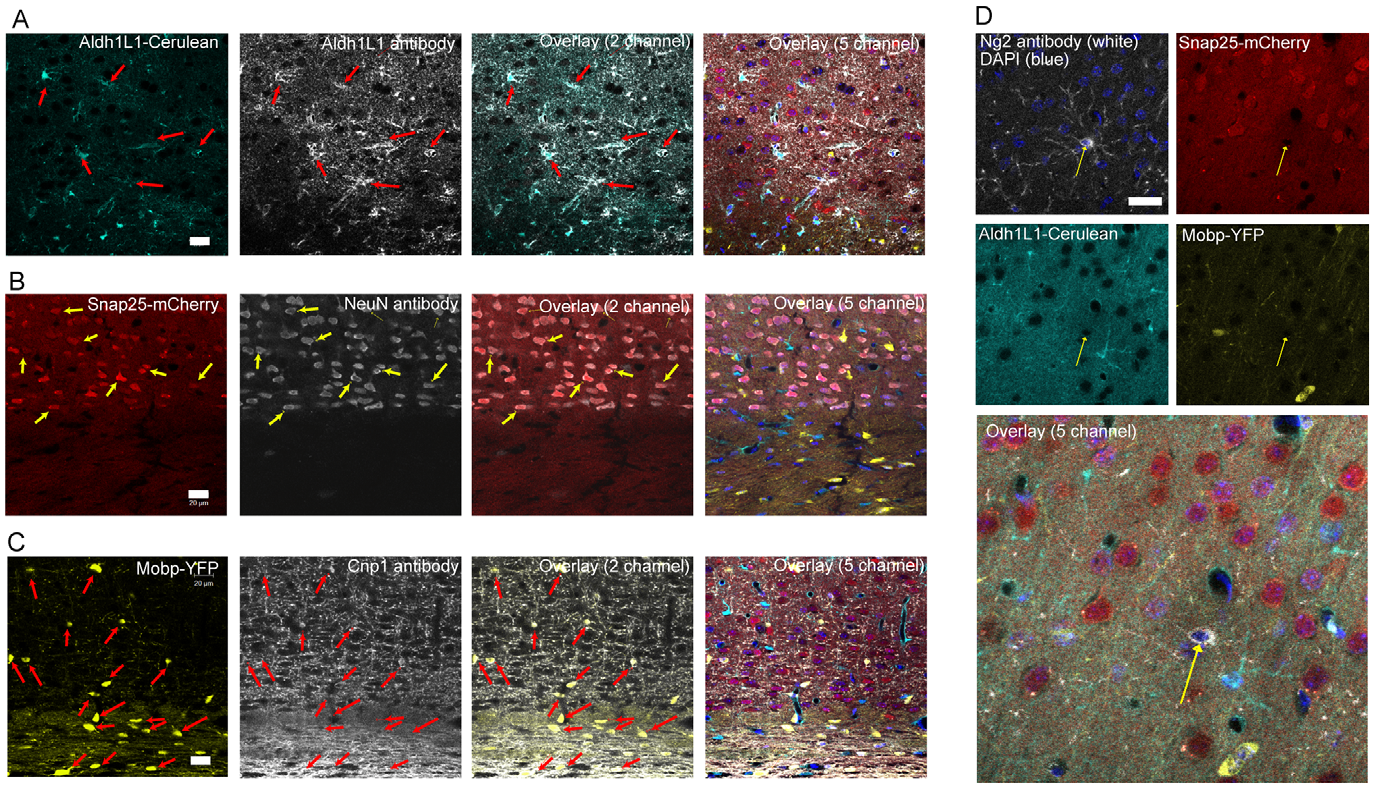
Immunofluorescence confirms Cerulean, mCherry, and YFP transgenes are each expressed in a distinct cell type. Combined confocal fluorescence (Cerulean, light blue, mCherry, red, YFP, yellow), immunofluorescence (primary antibodies detected with far red secondary antibodies, white), and two-photon (DAPI, nuclei, dark blue) microscopy from cortex, just above corpus callosum, on adult mice. A) Colocalization of *Aldh1L1*::Cerulean-Myc (light blue), with all cells (red arrows) immunofluorescent for Aldh1L1 protein (white). B) Colocalization of *Snap25*::mCherry (red), with all cells (yellow arrows) immunofluorescent for NeuN nuclear protein (white). C) Colocalization of *Mobp*::YFP-HA(light blue), with all cells (red arrows) immunofluorescent for Cnp1 protein (white). D) Lack of colocalization of any fluorophore with Ng2+ (white) cells (yellow arrow).

We focused first on the line carrying all three transgenes (Prism JD1849). Montage images of confocal fluorescent images reveal that the overall gene expression pattern of each fluorophore (Fig. 2A–C) corresponds largely to the DAB stained eGFP pattern evident in the corresponding single transgenic bacTRAP (eGFP-L10a) and GENSAT (eGFP) lines (Fig. S1). High power confocal fluorescent imaging in multiple regions revealed labeling in three distinct and non-overlapping cell types with the clear morphology of astrocytes, oligodendrocytes, and neurons (Fig. 2E–G, Fig. S1). To validate this morphological distinction, we undertook five color immunofluorescent study utilizing far-red secondary antibodies (Alexa 633 & 647) to visualize antibodies against markers of the various cell types, and DAPI for a nuclear counterstain to facilitate cell identification and counting (Fig. 3). For astrocytes we labeled with antibodies against the Aldh1L1 protein, which is considered pan-astrocytic [6], [25], as well as the traditional marker GFAP *(not shown)*. For neurons, we co-labeled with antibodies to the neuronal antigen NeuN (Fig. 3B). For oligodendrocytes, we co-labeled with the marker Cnp1 (Fig. 3C). In all cases we see marker overlap with, and only with, the appropriate fluorophore. Note that Mobp is not expected to be expressed in the Ng2+ oligodendrocyte precursor [27] and indeed we see no overlap of any fluorophore with glial cells immuno-positive for Ng2 (Fig. 3D).

The analysis above depended on the epi-fluorescence of each fluorophore. While the YFP-HA, and especially the mCherry, were quite bright, the Cerulean-Myc was dimmer. To determine if there was any trace mis-expression from this fluorophores, we also tested antibodies against Myc, and detected them with far red secondary antibodies. Myc antibodies labeled the appropriate cell type, and showed no trace mis-expression in either of the other cell types. In total, we found no cellular co-expression of any fluorophores in any region, demonstrating the independent regulation of each transgene.

**Figure 4:**
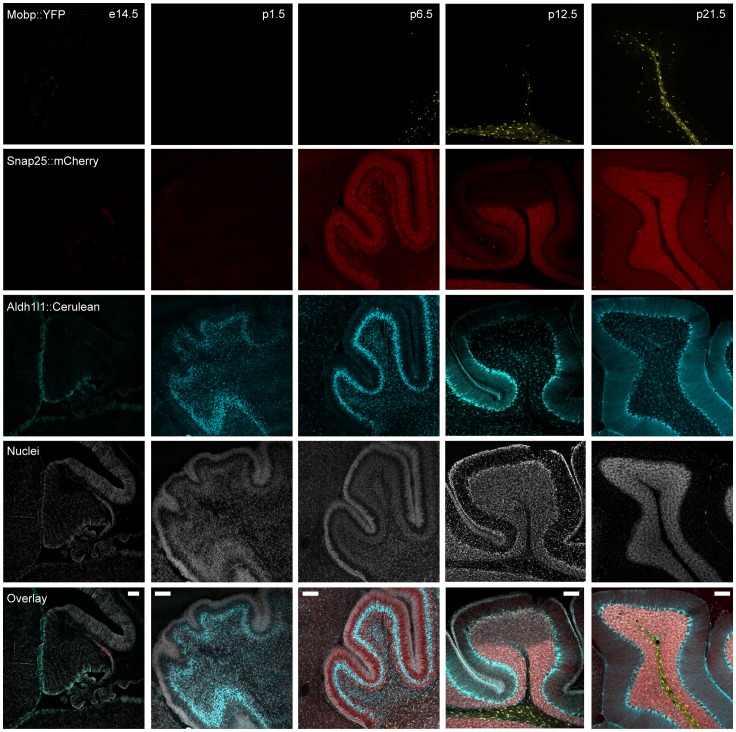
Cerulean, mCherry, and YFP transgenes each show independent temporal expression. Confocal fluorescent microscopy of Prism JD1849 mice cerebellums at five different stages of development shows a distinct ontogeny for each fluorophore, consistent with the known developmental expression patterns of the endogenous genes Snap25, Aldh1L1, and Mobp. *Colors as* *Figure 3* *. Scale bars – A–D, 20 microns, E, 100 microns.*

Finally, to test the potential utility of this line for *in vivo* imaging studies of development, and to further validate the accuracy of each transgene across a variety of ages, we studied the expression of these three transgenes across several stages in the cerebellum (Fig. 4). Each transgene showed a distinct developmental pattern of expression, with *Alhd1L1::Cerulean-Myc* being detectable at E14.5, consistent with its expression commencing in radial glia shortly after the beginning of gliogenesis. *Mobp::YFP-HA* appears early in the postnatal period, proceeding in a caudal to rostral manner, in a pattern consistent with the onset of myelination in mice. Signal is brightest around four weeks, consistent with the known expression of *Mobp* itself [28]. Finally trace *Snap25::mCherry* is detectable even as early as E14.5, though signal increases dramatically with the differentiation and maturation of cerebellar granule cells, consistent with the expression of a gene important for the function of axon terminals. Note that detector gain has been lowered substantially across development for mCherry in order to prevent detector saturation as signal becomes brighter with age (Fig. S2A). Indeed, by P14, *Snap25::mCherry* levels are robust enough to be visible macroscopically (Fig. S2B) in the cerebellum.

### All Transgenes are Co-inherited in a Single Locus

**Figure 5:**
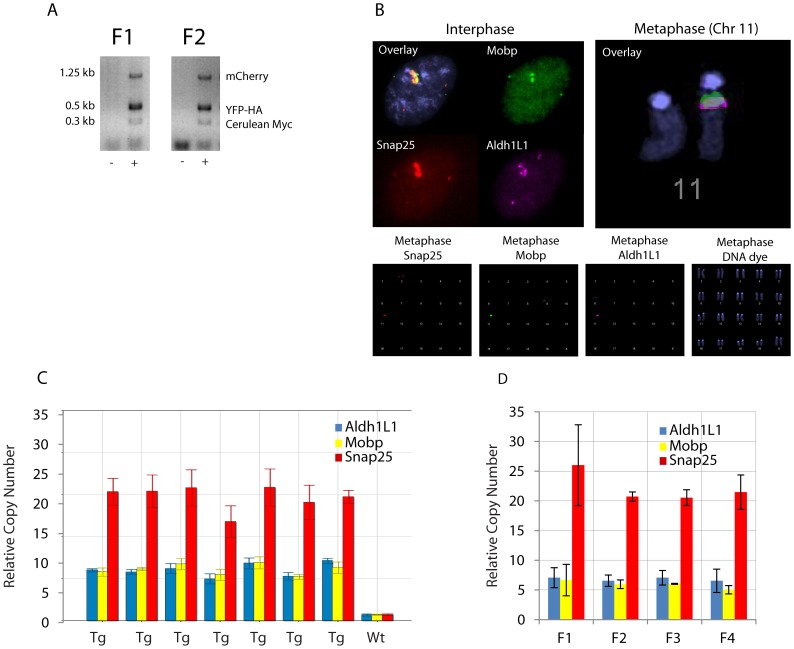
Cerulean, mCherry, and YFP transgenes are stably co-transmitted in a single locus. A) Representative PCR products from genotyping reactions of F1 and F2 progeny show co-inheritance of all three transgenes. B) Chromosomal Fluorescent In Situ Hybridization with BACs for Snap25(red), Aldh1L1(purple), and Mobp(green), shows separate signal in endogenous location for each gene(at 2qF, 6qG, and 9qF, respectively), and colocalization in a single transgenic locus at chromosome 11qA, in both metaphase(right, and bottom row), and interphase(left). C) qPCR on genomic DNA for Aldh1L1, Mobp, and Snap25, show an apparent consistency of trangene copy number within a litter of transgenic(Tg) animals, (*ddCT method normalized to Actb, single animals measured in triplicate, all probes scaled widltype animal, error bars standard deviation of technical replicates*). D) Across four generations, transgene copy number does not change significantly for any of the three transgenes (*p>.5 Snap25, p>.7 Mobp, p>.9 Aldh1L1, ANOVA, n = 2 mice per generation*). *All error bars standard deviations of biological replicates.*

In the absence of co-transmission, independent and accurate expression of three transgenes in a single mouse would be of limited utility. We designed independent PCR based genotyping assays for each of the transgenes (Fig. 5A). We genotyped twenty-two litters of mice (185 animals) across four generations and never failed to see complete co-transmission of all three transgenes (Fig. 5B). We have examined the anatomy of four additional complete litters of mice, and have never failed to see the appropriate expression of all three transgenes. This pattern of inheritance strongly suggests all three transgenes are in the same locus. To confirm this, we cultured astrocytes from the brain of Prism JD1849 mice and conducted a chromosomal FISH analysis using the unmodified BACs for *Snap25*, *Mobp,* and *Aldh1L1* as probes. Both metaphase and interphase FISH shows all three transgenes integrate into a single locus on chromosome 11, distinct from the endogenous loci of the driver genes (Fig. 5C). Thus both FISH and genotyping confirm a single locus for these transgene.

To determine the stability of the copy number across littermates and generations, we utilized qPCR assays for each transgenic locus. Copy number was found to be around 6–8 for *Aldh1L1::Cerulean-Myc,* 6-8 for *Mobp:YFP-HA,* and >20 for *Snap25:mCherry.* We did not see substantial variability of copy number within a litter (Fig. 5D), or a significant change in copy number across generations (Fig. 5E), suggesting copy number is as stable for these multiple insertions as for standard BAC transgenics [15]. It is worth noting that with the double transgenic line Prism JD1861, of ∼100 genotyped pups, we did have one anatomically confirmed pup with loss of YFP-HA from the locus. This suggests that recombination can occur at loci of BAC integration, but perhaps with no greater probability than the ∼1% per megabase typically found throughout the genome during meiosis.

### Efficiency of Transgenesis

To establish the overall efficiency of the method, we then attempted an additional twenty-five co-injections with a variety different (non-fluorescent) BACs and compared efficiency to ongoing single BAC injections at the time. All injections of both categories identified at least one transgenic pup integrating BAC DNA. Efficiency of identification of double transgenic pups (an average of 14% of pups born from a litter of injected eggs, n = 25 litters) was lower than corresponding single transgenics (23%, n = 24) during this period though still high enough to be practical for most applications. Unlike the Prism lines, independence of expression of these non-fluorescent BACs could not be easily evaluated, however most integration events did appear to occur at a single locus: The vast majority of these pups consistently co-transmitted both BACs to their offspring, consistent with integration of both BACs at a single locus.

### Shortcomings

**Figure 6:**
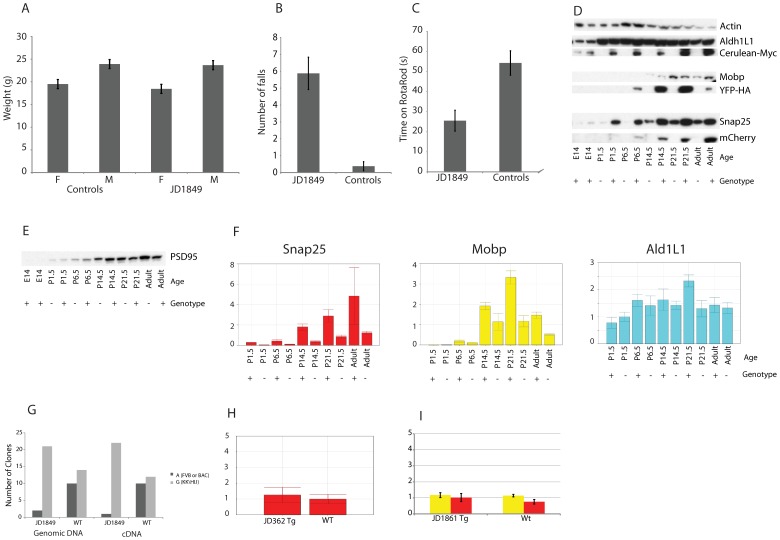
Prism JD1849 mice have abnormal behavior and Snap25 overexpression. A) Weights of adult mice are no different from age, sex and strain matched controls. Performance on rotarod is extremely poor: B) JD1849 mice fall or jump off the rotarod more often than matched controls, before apparatus is even turned on. (*p<3.5E-5, paired T-Test, n = 8 per group.* C) Once habituated enough to stay on apparatus, JD1849 mice still perform significantly worse than matched controls (*p<7.5E-05*). D) Western blots with antibodies to Aldh1L1, Myc, Mobp, HA, Snap25, and mCherry across development. *Actin is shown as a control for equal loading of the pairs within each time point. Total amount of protein loaded in each lane was equal across all time points.* E) Western blot for PSD95, shows no difference between wild-type and transgenic mice. F) qRT-PCR on RNA from brains of JD1849 mice shows excess Snap25 mRNA relative to littermate controls across time (*p<.04, Paired T-test*, *two-tailed, n = 10*). Aldh1L1 and Mobp were not significantly increased. *ddCT method, normalized to Actb. Error bars standard deviation of technical replicates.* G) Genotyping of cloned PCR products from brain cDNA and genomic DNA of F1 crosses to strain KK/HIJ. There is an over-representation of the G allele in cDNA (*Chi-square test, p<1.2E-5)*, suggesting excess Snap25 RNA may be coming from the BAC. H) Lack of apparent overexpression of Snap25 RNA in a Snap25 bacTRAP mouse brain from line JD362. *Error bars standard deviation of technical replicates.* I) qRT-PCR reveals no significant overexpression of either Snap25 or Mobp RNA in a line double transgenic for Snap25 and Mobp (*JD1861),* compared to littermates (*p>.05, T-test, one-tailed, n = 3 tg, 4 wt). Error bars standard deviation of independent biological replicates.*

The triple colored Prism mice described above provide support for our approach to generating mice with independently regulated transgenes expressed from a single locus. While these mice may already have applications for *in vivo* imaging of these cell types during development, or high-throughput microscopic characterization of mutant mouse lines, there were three unforeseen shortcomings of these mice, primarily regarding *Snap25::mCherry*. First, while mCherry has great spectral properties, it also has an apparent tendency to aggregate *in vivo* (Fig. S2A) and [14], which may lead to some toxicity [29]. Second, we failed to consider the density of axons and dendrites within the grey matter of the brain: an mCherry filled neuronal cell body is not easily distinguished from the surrounding field of mCherry filled neurites in many structures, such as cortex (Fig. 1F). While this can be rectified in counting applications in postmortem tissue by using a nuclear stain and counting overlap of fluorophores with the nuclei (Fig. 2), it may pose challenges in some structures for *in vivo* imaging. Finally, the mice themselves have an overt behavioral phenotype perhaps due to the mcherry aggregation, or to a marked Snap25 overexpression (Fig. 6, Movie S1 & S2), which would preclude their utility for applications that are sensitive to these features. Therefore, we first sought to determine whether the Snap25 overexpression was a typical consequence of our method, and then undertook a second round of transgenesis, both to correct these shortcomings, as well as to establish the reproducibility of the approach.

We first noted Snap25 overexpression while confirming the temporal accuracy of transgene expression via western blot (Fig. 6D). While each transgenic protein clearly has a temporal time course consistent with the endogenous protein, there is also an apparent excess of Snap25 protein across ages in transgenic mice compared to littermate controls. It is possible that excess Snap25 protein is due to either an abnormal number of synapses or neurons, or as some non-specific consequence of mCherry aggregates in neurons (Fig. S2a). However, Western blot for another synaptic protein, PSD95, shows no difference between wild-type and transgenics in the same samples (Fig 6E), arguing against these possibilities. Rather, the abundance of protein seems to be a consequence of a significantly elevated Snap25 mRNA level (Fig. 6F). To determine if this RNA comes from the genome or the Snap25 BACs, JD1849 transgenic mice (FVB background) were crossed once with the KK/HIJ strain. There is an A->G single nucleotide polymorphism (SNP), different between KK/HIJ strain (A) and FVB or BAC DNA (G), in the 3′ UTR of Snap25. Therefore, we PCR amplified this region from either genomic DNA or brain cDNA of F1 progeny, cloned these products into bacteria, and sequenced individual colonies. A and G alleles were counted. In wildtype littermates, both loci are approximately equally represented in genomic DNA as expected, and cDNA, suggesting both FVB and KK/HIJ alleles of Snap25 are equally transcribed into mRNA. In transgenics, there is an over-representation of the G allele in genomic DNA, as expected due to the presence of this allele on the Snap25 BAC. Surprisingly, there is also a significant over-representation of the G allele in cDNA as well, suggesting excess Snap25 RNA may be coming from the BAC in this mouse line (Fig 6G).

As Aldh1L1 BAC had no apparent overexpression (Fig. 6D,F), this is unlikely to be a general feature of multiplexing BAC transgenesis. Lack of overexpression of Snap25 RNA in a brain from a Snap25 bacTRAP line, made with the same BAC, but different transgene, suggests overexpression of a targeted BAC locus is not a general feature of BAC transgenesis, or the Snap25 BAC in particular (Fig. 6H). Finally, qRT-PCR reveals no significant overexpression of either Snap25 or Mobp RNA in a line double transgenic for Snap25 and Mobp (JD1861) (Fig. 6I). This suggests overexpression of SNAP25 is not a general consequence of having multiple BACs in a single locus, and that future lines utilizing the same BAC would be unlikely to demonstrate the same problem.

### Generation of Prism 2.0 Mice

To establish the reproducibility of the approach, and to overcome the shortcomings above, we undertook an additional round of transgenesis. For neurons, we modified a Snap25 BAC with a fusion of YFP-HA and the L10a ribosomal protein, which served to restrict most of the fluorophore to the cytoplasm and nucleolus, where most ribosomes are found [6]. For astrocytes we modified a *Aldh1L1* BAC with DsRedMax, which has similar spectral properties to mCherry, but has been engineered to reduce aggregation [29]. For oligodendrocytes, we modified a BAC for Mobp with Cerulean-Myc. Transgenesis was carried out as before.

**Figure 7:**
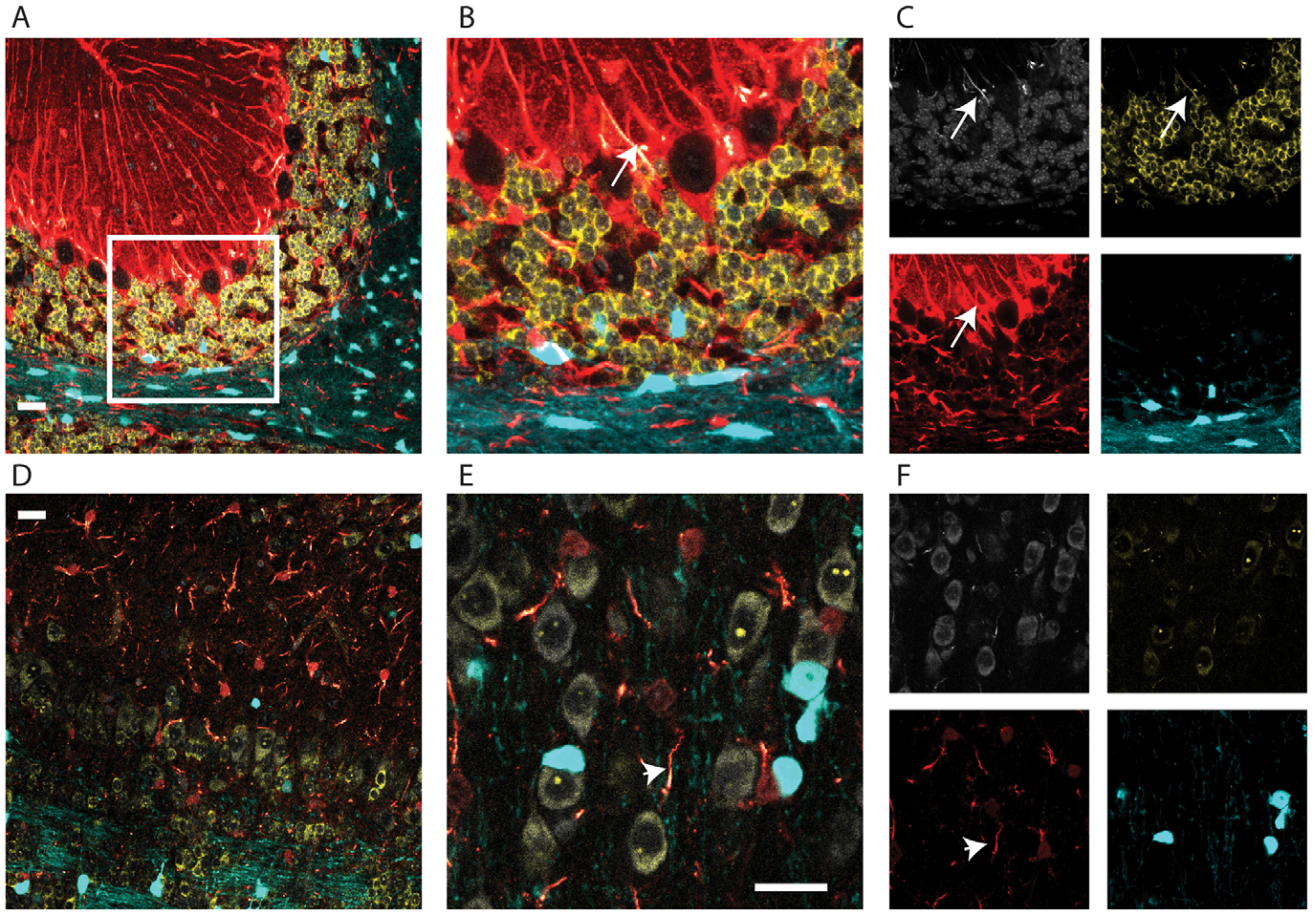
Multiple cell populations can be targeted reproducibly with distinct transgenes. A) Confocal fluorescent microscopy in cerebellum from a P24 mouse shows distinct expression of Snap25::YFP-HA-L10a (*yellow*), Aldh1L1::DsRedMax (*red*), and Mobp::Cerulean (*blue*), in neurons, astrocytes, and oligodendrocytes, respectively. Nucleic acid stained with To-Pro-3-Iodide (*white*). B) Higher magnification of region shown by box in A. C) Single channel images from B. Note that DsRedMax is of sufficient intensity in large caliber fibers to bleed through to white and yellow channels (arrow). D) Distinct cell types are also labeled in olfactory bulb and E) prefrontal cortex, though neurons are somewhat dimmer. F) Single channel images from E. Note that large caliber fibers are the most brightly fluorescing aspect of astrocytes (arrow head). All images in Fig. are from same brain section with same settings. *All scale bars 20 micron.*

From this second round of injection, we again identified a triple transgenic mouse line (Prism JD1989). As expected, this line has red astrocytes, blue-green oligodendrocytes, and yellow neurons (Fig. 7). With the YFP restricted primarily to the cell body and nucleolus, individual neurons are more easily distinguishable. While it does have a lower birth weight and some corresponding early post-natal mortalities, this line does not show the overt behavioral phenotypes of JD1849. This result suggests that multiple integration into a single locus, but with independent regulation, occurs frequently enough to be practical as a general solution to the allele problem.

### Availability

We have generated two triple transgenic lines showing genetic labeling of three distinct cell types. These lines can be visualized with relatively standard microscopy, and should provide a resource for the scientific community. These lines have been donated to Jackson Laboratories to facilitate their distribution (stock numbers: 018067 and 018068).

## Discussion

We believe that some improvements to the methodology and the mice could be made. First, while the glia are extraordinarily bright, the neurons in *JD1989* are relatively dim (Fig. 7). Second, while DsRedMax shows improvement over mCherry in the lack of obvious nuclear aggregates, two drawbacks remain: excitation/emission spectra are somewhat wider for this fluorophore [29], resulting in some spectral overlap in microscopy (Fig. 7C), and the fluorophore has a marked tendency to collect in the large caliber fibers of the astrocytes (Fig. 7E), resulting in a less complete view of the fine morphology of this cell type than with Cerulean (Fig. 3) or Egfp-L10a [6]. (Also, Aldh1L1 is also highly expressed in the liver, and perhaps the presence of these fibers there contributes to the low birth weight and early mortality). Third, the behavioral phenotypes (JD1849), and postnatal lethality (JD1989) likely preclude the use of these mice for many *in vivo* applications, though not necessarily for studies of development, *in vitro* differentiation and lineage tracing. Future experiments optimizing relative amounts of BACs injected, and experimenting with other fluorophores, could lead to lines with more even expression, and without detrimental phenotypes.

Overall, the long term stability of the BAC loci seems sufficient. While we have some evidence from the JD861 line that meiotic recombination can occur, our data do not suggest that these double and triple transgenics are any more unstable than would be expected for any other locus in the genome. In fact, it may be equally possible to take advantage of meiotic processes to *increase* BAC copy number: paired chromosomes with large duplicated regions provide an efficient substrate for non-homologous allelic recombination (NAHR) [30]. If homozygote BAC mice are generated (by simply breeding the heterozygotes), during meiosis in their germlines, NAHR at the paired BAC loci would allow for potential increases in the overall BAC copy number in heterozygote progeny. This approach may provide a method for ‘dialing up’ or ‘dialing down’ copy number of BAC lines after their generation, and may be useful to increase or decrease transgene expression level, particularly for single transgenic BAC mice.

Regardless, we have demonstrated here a simple solution to the allele problem by simultaneous co-injection of multiple BACs, permitting multiple transgenes to be expressed from the same loci, but with independent regulation. Further optimization of injection conditions and DNA preparation may improve efficiency of transgenesis, though already the current methodology appears sufficient for many applications. Using a different set of non-fluorescent transgenic constructs, we attempted over twenty-five injections for double BAC transgenic lines, and have successfully identified double co-integrates for all. None of these lines have overt abnormalities of birth weight or behavior. Having established that the method is feasible for simultaneous labeling of distinct cell types, it will be even more interesting to apply it to simultaneous experimental manipulation of genetically labeled groups of cell types *in vivo* [8], [9], [20], [21].

## Materials and Methods

### BAC Modification

New shuttle vectors were constructed by removing the eGFP-L10a from the s296 vector [6] and replacing it initially with mCherry [24], Cerulean [23] with a PCR cloned C-terminal Myc tag, YFP [22] with a PCR cloned C-terminal HA tag, DsRedMax [29], or PCR cloned YFP-HA-L10a transgene. The three prime Myc tag was added to Cerulean and HA tag to YFP so that these two GFP derivatives could be distinguished easily by different antibodies. BACs covering Snap25 (RP23-290A18), Mobp (RP23-172H7), or Aldh1L1 (RP23-7M9), were modified in bacteria using standard methods [31], and proper recombination was confirmed with southern blotting and BAC fingerprinting. DNA was purified using CsCl gradient ultracentrifugation. BAC DNA was dialyzed into injection buffer (10 mM Tris, 0.1 mM EDTA, 100 nM NaCl, Ph 7.5) using MF membrane filters (Millipore) and concentration and integrity was measured with pulse field gel.

### Transgenesis, Animal Husbandry and Histology

All procedures involving animals were approved by the Rockefeller University Institutional Animal Care and Use Committee.

Equal amounts of the three DNAs were mixed together and diluted for a final total DNA concentration of 1 ng/µl, then injected into mouse eggs using standard methodology [31]. Founders were identified with PCRs specific for each transgene, and line was maintained by crossing to wild-type FVB at each generation. Because of behavioral difficulties, only female *JD1849* transgenics were used for breeding, and an additional wild-type female c57/bl6j mouse was included in the breeding cage to provide some degree of surrogacy. Transgenic progeny were identified from amongst the white pups by PCR as above.

For histology, mice were euthanized and perfused transcardially with PBS followed by 4% paraformaldehyde in PBS, and brains were dissected and postfixed in 4% paraformaldehyde. Embryonic mice were dissected from timed-pregnant females and immersion fixed in 4% paraformaldehyde. All tissues were cyroprotected in 30% sucrose PBS, and cut to 40 micron floating sections (p7 or older) or 20 micron sections affixed to Superfrost Plus slides (younger ages) on a Leica cryostat. For immunofluorescence, sections were blocked with 5% normal donkey serum in 0.25% Triton X-100 in PBS for thirty minutes, and then exposed to primaries overnight, washed in PBS, and exposed to appropriate Alexa dye conjugated secondary antibodies and fluorescent nuclear counterstains (DAPI or To-Pro-3-Iodide). Primaries included GFAP (Dako), Aldh1L1(Dr. Sergey Krupenko), NeuN, Ng2, Cnp1 (Chemicon), Myc (Upstate Biotechnology), and HA (Roche).

### Cell Culture and Transfection

Fluorophores were cloned from shuttle vectors into expression vector pEGFP-C1 (Clontech) in place of eGFP and transfected using Lipofectamine 2000 following manufactures protocols into 293T cells plated on glass coverslips. 48 hours later, coverslips were washed in PBS and fixed in 4% paraformaldehyde PBS, and mounted with Prolong anti-fade kit for microscopy.

### Microscopy

All images were taken with a LSM 510 NLO inverted multiphoton system. For four color imaging, system was utilized as a standard confocal: YFP-HA and YFP-HA-L10a were excited with a 514 nm laser, and detected with a Band Pass 540/20 filter in place. mCherry and DsRedMax were excited with a 543 nm laser and detected with a 565-615 IP Band Pass filter. Cerulean-Myc was exited with a 458 nm laser, as detected with a 470-500 Band Pass filter. Far red Alexa dyes and To-Pro-3-Iodide were excited with a 633 nm laser and collected with the Lambda mode of the Zeiss system. For five-color imaging, system was utilized as above plus DAPI was visualized with a coherent chameleon 2 photon system. Microscope settings were optimized using 293T single and triply transfected cells (Fig. S1B).

### SDS PAGE and Western Blotting

Whole brains from wildtype and transgenic littermates were harvested at the indicated time points. Animals from P1 to P21 were all from same litter. Brains were homogenized with Teflon homogenizers in 3-detergent Lysis buffer (50 mM Tris Cl, Ph 8.0, 150 mM NaCl, 0.1% SDS, 0.02% Na Azide, 0.5% Na Deoxycholate, 1% NP-40), in the presence of a protease inhibitor cocktail (Roche), incubated for 5 minutes at 4 C, sonicated for 15 seconds, and spun at 20,000 g for thirty minutes. Supernatant was kept. Protein was quantified with BCA Assay (Pierce) per manufacturer’s instructions. 20 ugs of each sample was loaded on parallel gels for SDS page using the Invitrogen NUPAGE gel system, following manufacturer’s instructions. Protein was transferred to PVDF membranes(Polyscreen, Perkin Elmer) using semi-dry transfer apparatus (BioRad), following manufacturer’s instructions. Membranes were blocked with 5% Powdered Milk (Carnation) in PBS with 0.1% Tween, then incubated in the same buffer with the primary antibodies above, as well as Snap25(Abcam), Mobp(Santa Cruz Biotechnology), RFP(Dsred, Clontech). Primaries were detected with HRP conjugated secondary antibodies, and Super-Signal West Femto substrate (Thermo Scientific). Each blot was exposed for several different durations. Each band was found at the predicted size, and there were no extraneous bands for any proteins, except for two: a 25 kd band present in wildtype and transgenic samples in the ALdh1L1 western, and an extra band the size of the endogenous Myc protein was detected in embryonic samples with the Myc western.

### Quantitative PCR and Reverse Transcription Quantitative PCR

To measure copy number, tail tips were digested overnight in lysis buffer (50 mM Tris, 1 mM EDTA with.05% Tween and Proteinase K, Ph 8), and DNA was prepared with phenol chlorofom extraction followed by ethanol precipitation. DNA was resuspended in TE and carefully diluted to 10 ng/µl. 20 ngs were used in qPCR assays. Data were normalized to B-actin utilizing the ddCT method. For relative copy number across generations, each BAC primer set was further normalized to an arbitrary transgenic sample.

For gene expression analysis, RNA was harvested from transgenic and matched controls using Trizol (Invitrogen), following manufactures instructions, and treated with Turbo DNAse I (Ambion), to remove trace genomic DNA. cDNA was synthesized from 1 µg of total RNA using Protoscript Reverse Transcriptase (New England Biolabs), primed with Oligo d(T)23VN following manufacturer’s instructions cDNA was purified with Qiaquick columns (Qiagen), eluted in 200 µls, and 1 µl was used for each reaction.

For all qPCR assays: all assays were performed in triplicate, using Maxima SYBR Green qPCR Master Mix (Fermantas). Conditions were 35 cycles of 95 C, 30 Seconds, 63 C, 30 seconds, and 72 C, 30 seconds, followed by a standard melt curve. Products were sequenced to confirm specificity of reaction.

### Behavior

Three male and five female, adult Prism JD1849 mice, and age and sex matched FVB controls, were assayed on a Rotamex Rotarod (Columbus Instruments) [Start speed 0, End Speed 30, Acceleration Interval 4, Speed 1, Duration 120] for consecutive 10 trials, with a 30 second rest between trials. Also quantified was the number of times the mice fell off rotarod before apparatus could be turned on for first trial.

### Fluorescent In Situ Hybridization

BAC DNA was directly labeled by nick translation using fluorescent nucleotides from Abbott Molecular: Green-dUTP (RP23-172H7), Orange-dUTP (RP23-290A18), and Red-dUTP (RP23-7 M9). Metaphase preparations were made from cultured astrocytes according to standard cytogenetics procedures following Colcemid treatment at 0.05 µg/ml for 40 minutes. 50-70 ng of each probe, plus 4.5 µg mouse Cot-1 DNA, were hybridized to metaphase slides according to standard FISH procedures. Images were captured using a MetaSystems FISH workstation, comprising a Zeiss Axioplan microscope and motorized stage controlled by Isis software.

### Statistical Analysis

For behavioral analysis: for each mouse, data were averaged across all trials, then groups were compared using Paired T-Test utilizing the SPSS software package.

For copy number qPCR (Fig 5E): Technical replicates were averaged and the unit of statistical analysis was independent biological replicate (mouse). For statistical analysis a single factor ANOVA was run for each transgene in MS Excel (Analysis Tool Pak). No significant effect was found for mouse generation (p>.50 for all tests).

For qRT-PCR: Replicates treated as above. A two tailed T-Test, paired on age, was used to compare expression of transgenic to wildtype littermates mice across time (Figure 6F) A one tailed T-Test was used to test for overexpression in JD1861 mice (Figure 6I). Both were calculated in MS Excel.

For allelic expression imbalance: a Chi Square test statistic was used to determine if WT or JD1849 cDNA deviated from an expected ratio of 1∶1 A:G. Calculated in MS Excel.

## Supporting Information

Figure S1 **Triple transgenic mouse line successfully recapitulates spatial expression pattern of three individual lines in cerebellum.** GFP fluorescence images from three transgenic lines of the GENSAT project show three different patterns of expression in cerebellum A) Snap25::GFP is found in cell bodies of Granule Cell Layer(GCL), and axons in Molecular layer(ML), but with little or no signal in the white matter (WM), as is Snap25::mCherry in the Prism line. B) Both GENSAT Aldh1L1::GFP and the Prism Adl1L1::Cerulean show scattered expression in GCL and WM, and a line of cells (Bergman glia) at the GCL/ML division, which extend processes into ML. C) Both GENSAT Mobp::GFP and the Prism Mobp::YFP show expression densely in WM and in scattered cells in GCL, and no signal in ML. GENSAT images were only available from Adult (Snap25, Mobp) or P7 (Aldh1L1). Prism is shown at P12.5.
(TIF)

Figure S2 **mCherry expression increases dramatically with age.** A) P12.5 cerebellum imaged with identical settings as P6.5 cerebellum from Figure 2, shows saturation of mCherry signal (bottom panels, red pixels), indicating dramatic increase in cellular mCherry concentration. Also note bright mCherry puncta in neurons (red pixels, lower right panel), suggesting some aggregation of this protein *in vivo*. B) Whole mouse brains, viewed from caudal perspective shows an increase in pinkness corresponding to completion of cerebellar granule cell neurogenesis, which corresponds to increased mCherry fluorescence in the cerebellum (lower panels). C) Clear distinction of brain color between Prism JD1849 transgenic mice (Tg), and wildtype littermates (Wt) in adult brains.
(TIF)

Movie S1 **JD1849 mice have obvious behavioral abnormalities.** Abnormal activity of JD1849 mice in home cage. Two with black markings have just been returned to home cage.
(WMV)

Movie S2 **Other lines do not have obvious behavioral abnormalities.** Normal activity of control transgenic mice (JD1864) upon return to home cage.
(WMV)
