# Supplementary figures and images for: Mouse Transgenesis in a Single Locus with Independent Regulation for Multiple Fluorophores
Source: PLoS One. 2012 Jul 12;7(7):e40511. doi: 10.1371/journal.pone.0040511 (PMC3395707; doi:10.1371/journal.pone.0040511)

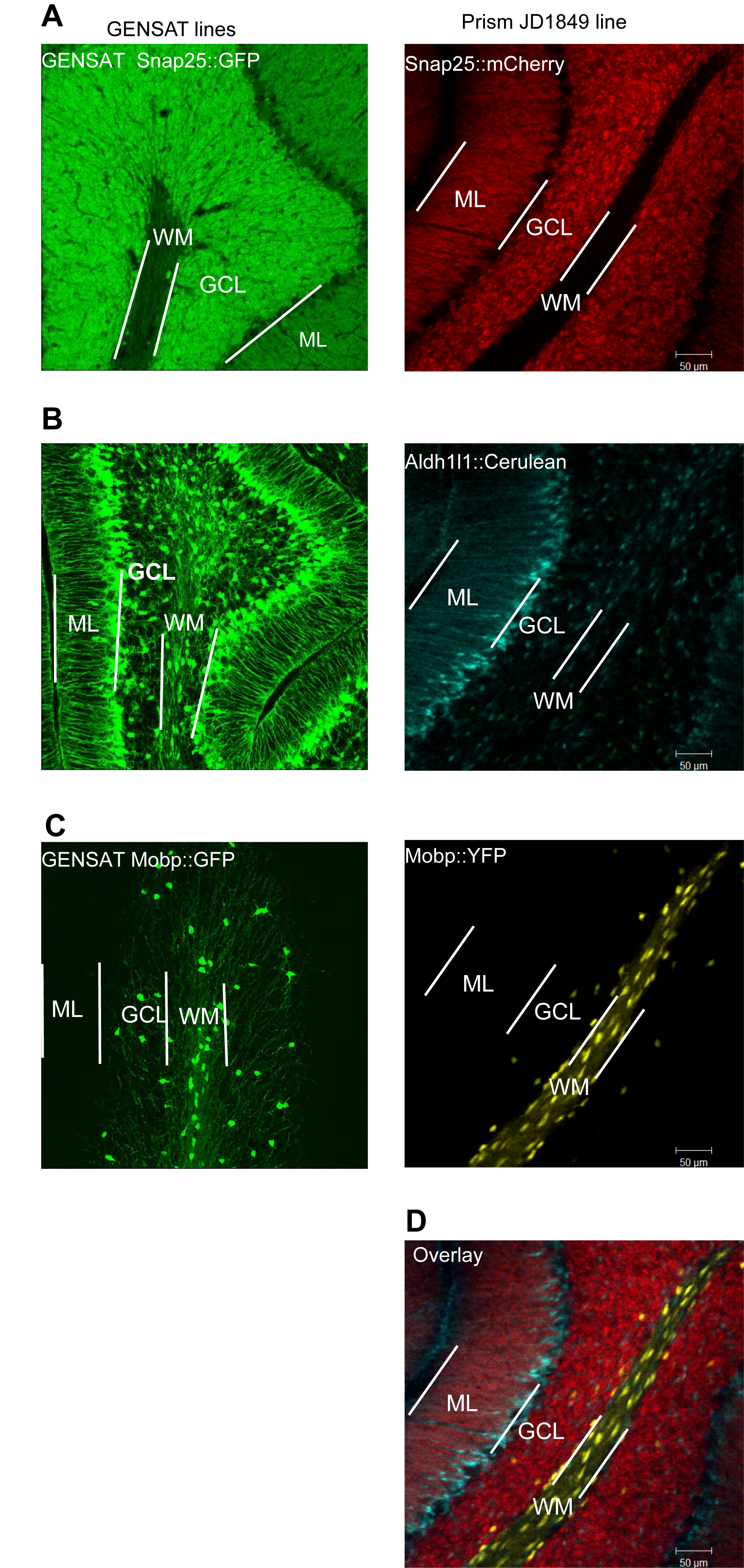

Supplement: Figure S1 — Triple transgenic mouse line successfully recapitulates spatial expression pattern of three individual lines in cerebellum. GFP fluorescence images from three transgenic lines of the GENSAT project show three different patterns of expression in cerebellum A) Snap25::GFP is found in cell bodies of Granule Cell Layer(GCL), and axons in Molecular layer(ML), but with little or no signal in the white matter (WM), as is Snap25::mCherry in the Prism line. B) Both GENSAT Aldh1L1::GFP and the Prism Adl1L1::Cerulean show scattered expression in GCL and WM, and a line of cells (Bergman glia) at the GCL/ML division, which extend processes into ML. C) Both GENSAT Mobp::GFP and the Prism Mobp::YFP show expression densely in WM and in scattered cells in GCL, and no signal in ML. GENSAT images were only available from Adult (Snap25, Mobp) or P7 (Aldh1L1). Prism is shown at P12.5. (TIF) [file pone.0040511.s001.tif]

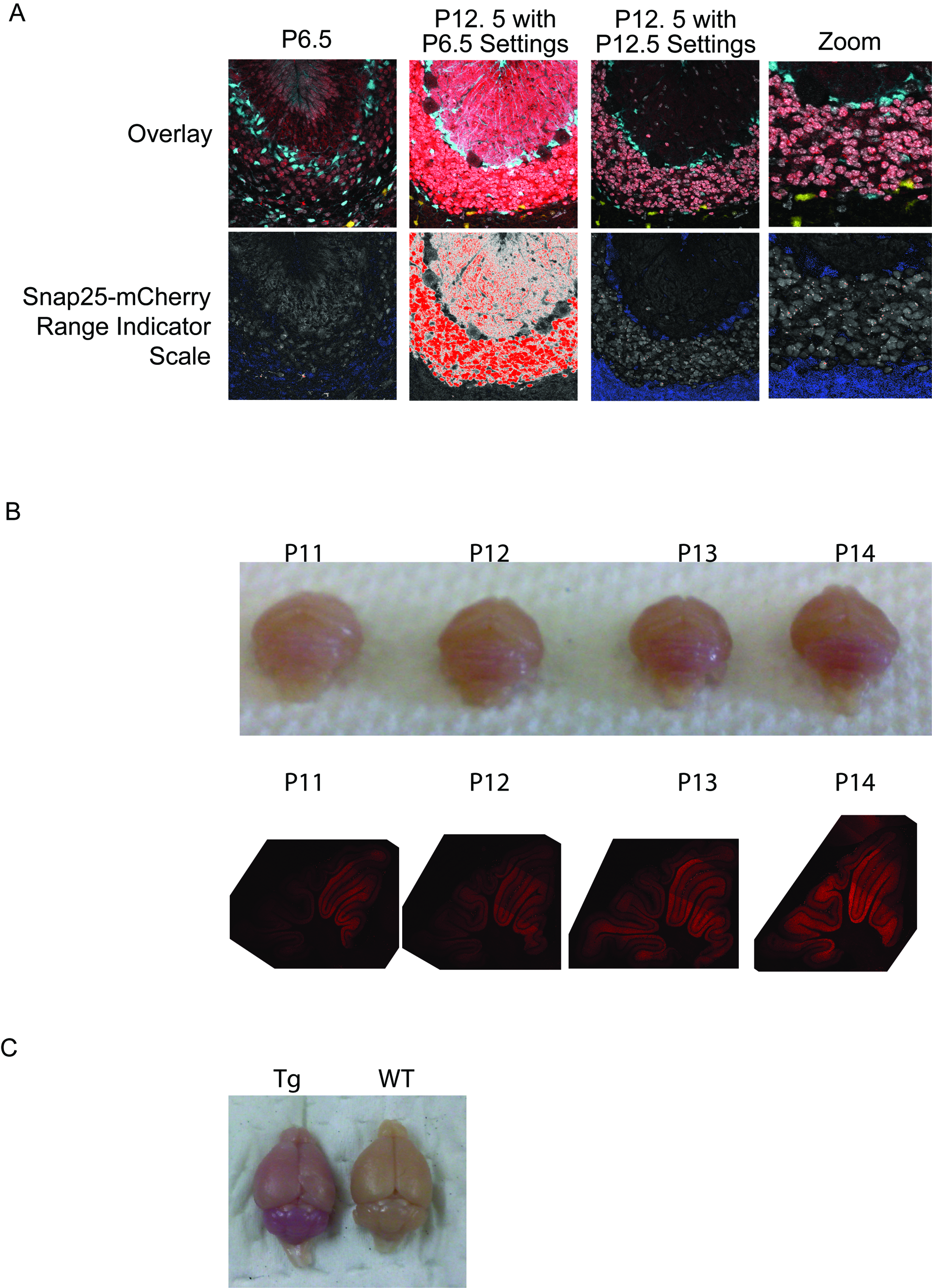

Supplement: Figure S2 — mCherry expression increases dramatically with age. A) P12.5 cerebellum imaged with identical settings as P6.5 cerebellum from Figure 2, shows saturation of mCherry signal (bottom panels, red pixels), indicating dramatic increase in cellular mCherry concentration. Also note bright mCherry puncta in neurons (red pixels, lower right panel), suggesting some aggregation of this protein in vivo. B) Whole mouse brains, viewed from caudal perspective shows an increase in pinkness corresponding to completion of cerebellar granule cell neurogenesis, which corresponds to increased mCherry fluorescence in the cerebellum (lower panels). C) Clear distinction of brain color between Prism JD1849 transgenic mice (Tg), and wildtype littermates (Wt) in adult brains. (TIF) [file pone.0040511.s002.tif]
